# Diarrhœa in Children

**Published:** 1884-07-20

**Authors:** Theophilus Parvin

**Affiliations:** Professor of Obstetrics and Diseases of Women and Children in Jefferson Medical College


### DIARRHŒA IN CHILDREN.

By THEOPHILUS PARVIN, M.D.,

Professor of Obstetrics and Diseases of Women and Children in Jefferson Medical College. Reported by William H. Morrison, M.D., for the Archives of Pediatrics, April 15th, 1884.

GENTLEMEN: The two cases first presented this morning illustrate a common affection, one that you will be often called to treat, diarrhœa in infants and in children after the usual time of weaning. Here is an infant six months old, pale, its face showing that it suffers, and while the child is not positively emaciated it is far from being as plump and well nourished as it should be. It is now six months old. Its mother died but a week or two after its birth, and since then it has been cared for by its grandmother, who brings it here. It has been brought up solely upon artificial food. Various kinds of food have been tried, and condensed milk finally adopted, as apparently the least injurious. Yet the condensed milk is far from being a good food in this case, since the child has at least twelve operations from the bowels every twenty-four hours, and, moreover, the operations cause a great deal of straining and pain; further, the grandmother tells us that after the straining a reddish lump appears: that is a prolapse of the rectum or, rather, of its mucous membrane, an affection which is not uncommon, while the former is exceedingly rare. The stools are not peculiarly offensive in odor, contain no blood, slime or mucus, and in color they are yellowish and green. Further, an examination of the buttocks and groins shows a violent erythema occupying these parts. The grandmother tells us that this has been present since the child was three weeks old, and every time the child urinates or has a stool, it cries with the suffering caused by the contact of these discharges with the raw, inflamed surfaces.

You at once see that neither as to frequency nor as to color are the discharges from the bowels normal. Such discharges should not occur oftener than two or three times in the twenty-four hours, and they ought to be of a light yellow color. Let me urge upon you the importance, in treating these diarrhœal affections of children, of knowing not only the actual number of discharges in a day, but also of seeing for yourselves, observing their quantity, consistence and color.

Now, in the treatment of this patient, the first thing is a complete change of diet. Let the condensed milk be abandoned, and let the child have, instead, cow's milk, diluted with one-third of an equal quantity of gum-arabic water, gelatine water, or albumen water. When the diarrhœa is better, barley water may be used for the diluent. As I have had occasion to say to you before, to dilute cow's milk with water is one of the worst of practices in infant feeding, for by such great reduction of the nutritive properties of the milk a double quantity of fluid must be taken, and thus indigestion from overloading the stomach or from too frequent feeding. Let me say still further as to artificial food for infants: It is impossible to say, in a given case, that one or that another kind of food will prove best. Have as admirable theories as you please in the selection and combination of ingredients, the final test is in experiment, and the experiment sometimes sets at naught the wisest, most scientific theories. Therefore, when you are directing the sort of diet an infant is to have, you are simply trying an experiment, and if the experiment is a failure don't persevere in it—do not try vainly to compel facts to correspond with your theories, but yield obedience to the facts, and try another experiment.

Now, as to the nature of the diarrhœa, it is simply an intestinal catarrh, not an inflammation, from which this child is suffering. Its chief, if not the sole cause, is the food that is given—not that such food may not prove best in some other children, but that it does not agree with this child, and food appropriate to its wants will improve, if not cure, the diarrhœa. The stools, when first passed, you remember, are partly green, and in the medicinal treatment of such case it is well to include antacids. If the stools become green after exposure to the air, no such indication in the treatment would be presented. I think it would be well, for the first few days, to give this infant an occasional dose of subnitrate of bismuth, prepared chalk and opium—about the thirtieth of a grain of the last, and two or three grains of each of the other ingredients. What shall be done for the erythema? Perfect cleanliness of the parts is of first importance, soiled napkins to be promptly removed, and none but perfectly clean ones applied in their place; then careful bathing and drying, after which thoroughly dust the surface with lycopodium. Do not use starch for this purpose, as it is liable to form, with the moisture that exudes from raw surfaces, hardened masses and cakes, which are irritating, and are not easily removed; but, if you choose, you may mix the lycopodium with an equal quantity of oxide of zinc; sometimes, by the way, an ointment of oxide of zinc proves better than any powder, applied to the surface. What is to be done for the rectal prolapse? With less frequent operations, the liability to this accident is lessened; but when it occurs, let the tumor be bathed in cold water, and then have the child kept upon its back, if possible, for a short time, the thighs being kept close together. In case of paralysis of the anal sphincter, cold astringent injections will prove useful, and its persistence in spite of these may be met by injections of warm water containing one drop of tincture of nux vomica and five drops of the fluid extract of ergot, one or two each day. A recent and very good recommendation for the tenesmus that frequently is a prominent symptom in this rectal prolapse, made by Dr. Droixhe—Journal d' Accouchements, January, 1884—is the introduction of a suppository composed of iodoform.

The second child now presented to you is twenty-three months old, and since it was fifteen months old it has been taking, according to its mother's statement, "Table-food," and that means that it has been taking a great deal that it ought not to have had. You will so often, when asking after the diet of even a younger child, have the answer, "Pretty much everythlng that is going," that one wonders, not that so many infants die, but that so many live, when parents set them to digging their graves with their teeth, sometimes, indeed, with their gums. You may judge that there is not the most remarkable wisdom manifested in feeding this child, for it can neither be charmed into silence by your faces or by my speech, but it is by munching a piece of pie, an article of food which is often to be credited with causing indigestion in adult stomachs, and which few men and women can eat with freedom and impunity. For two months the child has had diarrhœa, the evacuations sometimes being as frequent as eighteen in twenty-four hours; these are of a brownish color, contain no hard lumps, nor mucus or blood, and there is no prolapse of the bowel resulting from them. Further, this child has had fever occurring every other night, and for this quinine has been successfully given. There is a history of tuberculous disease in some of the near relatives, though not in any of the immediate ancestors. One of these relatives was brought to the dispensary suffering, it was thought, with entero-mesenteric tuberculosis, and, therefore, there is more anxiety as to this child.

The child's complexion is not clear, but that may be attributed to the malarial poisoning. Its countenance does not show the suffering which is often a marked characteristic of chronic tuberculous disease; nor are the discharges from the bowels such as occur in this disease: they are not yellow or grayish fluids mixed with lumpy masses and portions of undigested food. The conclusive evidence of tuberculous enteritis would be detected by the touch of the enlarged mesenteric glands. The point where you seek these is in the middle of the abdomen and below the umbilicus. Bouchut has called attention to the fact that fecal masses, scybala, may be mistaken for enlarged mesenteric glands, but the former are found at the sides, especially at the sigmoid flexure, while the latter have, as above stated, a median position.

In the treatment of this patient the first thing is to insist upon no more solid food, especially pie, being permitted. Let the child have a liquid diet, such as milk and the lighter animal broths, a daily bath and protection from cold by suitable clothing, and avoidance of exposure will be directed. The quinine will be continued, to guard against a return of the malarial attacks, and also as a useful tonic, and for the diarrhœa the officinal chalk mixture in a dessertspoonful dose, a little tincture of krameria and the camphorated tincture of opium being added to each dose, will be directed.—*Maryland Med. Journal.*

